# Evaluation of venous pathology of the lower extremities with triggered angiography non-contrast-enhanced magnetic resonance imaging

**DOI:** 10.1186/s12880-019-0395-4

**Published:** 2019-12-17

**Authors:** Yao-Kuang Huang, Yuan-Hsi Tseng, Chih-Hung Lin, Yuan-Hsiung Tsai, Yin-Chen Hsu, Shih-Chung Wang, Chien-Wei Chen

**Affiliations:** 1 Division of Thoracic and Cardiovascular Surgery, Chang Gung Memorial Hospital, Chiayi, Taiwan; 2 Wound Center and Plastic Surgery, Chang Gung Memorial Hospital Chiayi Branch, Chiayi, Taiwan; 3 College of Medicine, Chang Gung University, Taoyuan, Taiwan; 4 Department of Diagnostic Radiology, Chang Gung Memorial Hospital Chiayi Branch, Chiayi and Taoyuan, Taiwan; 5 Department of Medical Imaging and Radiology, Shu-Zen Junior College of Medicine and Management, Kaohsiung, Taiwan; 6 Institute of Medicine, Chung Shan Medical University, Taichung, Taiwan

**Keywords:** MRI, Non-contrast, Venography, TRANCE, Static ulcer, Venous disease

## Abstract

**Background:**

To explore the diagnostic performance of triggered angiography non-contrast-enhanced magnetic resonance imaging (TRANCE-MRI) for the evaluation of venous pathology of the lower extremity.

**Methods:**

This was a single-centre prospective cohort study of 25 patients with suspected venous disease in the lower extremities. Each patient received Doppler ultrasonography (for venous evaluation) before the scheduled TRANCE-MRI (for venous and arterial evaluations) on a 1.5 T MR scanner (Philips Ingenia, Philips Healthcare, Best, the Netherlands), followed by lymphography and computed tomography angiography that were arranged according to the diagnostic indications.

**Results:**

The sensitivity, specificity and accuracy of TRANCE-MRI were 85.7%, 88/9 and 88%, respectively. The inter-rater agreement for deep vein thrombosis (DVT) of the thigh between the ultrasonography and TRANCE-MRI results was substantial agreement (Cohen’s kappa κ, 0.72). In ultrasonography-negative cases, TRANCE-MRI detected four additional cases (16%, 4/25) of DVT; three cases (12%, 3/25) of venous compression caused by pelvic lymphadenopathy, hip prosthesis or knee joint effusion; one case (4%, 1/25) of vena cava anomaly; two cases (8%, 2/25) of occult peripheral artery disease (PAD); and one case (4%, 1/25) of an occluded bypass graft.

**Conclusion:**

TRANCE-MRI can be used as an alternative and objective tool for assessing lower extremity diseases, especially suspected venous pathology. Compared with ultrasonography, TRANCE-MRI plays a better role in assessing varicose veins of the lower extremities and deep veins of the pelvis and abdomen. However, false-positive results may occur in the left common iliac vein of elderly patients. Finally, occult PAD rarely occurs in patients with suspected lower extremity venous disease. Therefore, we recommend performing the TRANCE-MRV protocol instead of the full protocol (MRV + MRA) in the clinical setting in patients with venous scenarios.

## Background

Venous pathology of the lower extremities is a critical public health problem with economic and social consequences. It includes scenarios from minor varicose veins (VVs) and nuisance venous static ulcers (SUs) to potentially deadly deep vein thrombosis (DVT) [1–3]. Ultrasonography serves as the standard first-line tool for evaluating lower limb swelling. However, ultrasonography is both difficult and insensitive in patients exhibiting obesity, oedema, or tenderness who have undergone recent hip or knee arthroplasty as well as those with casts, bandages, or immobilization devices. Ultrasonography also does not adequately assess the pelvic region or the deep veins [4]. Previously, conventional venography was considered the standard tool for the detection of DVT in patients with VVs [5, 6]. However, this procedure is time-consuming and invasive and requires the use of contrast medium and radiation exposure. Contrast-enhanced computed tomography (CT) and magnetic resonance imaging (MRI) have been widely discussed and used in clinical practice. However, the complications associated with contrast agents are concerning and can be fatal. Nephrogenic systemic fibrosis (NSF) is a rare but severe complication of using gadolinium-based contrast agents in patients with renal insufficiency [7–9].

TRiggered Angiography Non-Contrast Enhanced (TRANCE) imaging is a non-contrast-enhanced MR technique that exploits the differences in vascular signal intensity during the cardiac cycle for subsequent image subtraction. It can provide high-resolution and background-removed blood vessel images that can display arteriography along, venography along or background-removed all blood vessels. Although previous studies have discussed some non-contrast-enhanced MR techniques (e.g., time-of-flight and phase-contrast), the research on TRANCE-MRI is still rare, and most studies focus only on its application in arterial disease [10–12]. To compensate for the lack of relevant research, we attempted to design a prospective study to explore the clinical utility of TRANCE-MRI in the assessment of lower extremity disease. To the best of our knowledge, this was the first study to apply TRANCE-MRI for the assessment of venous pathology in the lower extremities.

## Methods

### Subjects

The Institutional Review Board (IRB) of Chang Gung Memorial Hospital approved this study (IRB number: 201700389B0). We prospectively collected information from patients who underwent lower extremity examinations at the tertiary hospital vascular wound care centre from April 2017 to March 2018. Patients were eligible for inclusion in the study if they had a clinical indication for computed tomography angiography (CTA) for the evaluation of pelvic and leg vessels. The exclusion criteria were pregnancy and MRI contraindications (e.g., non-MRI-compatible device implants). In addition, patients with poor compliance and patients with multiple comorbidities that prevented them from lying down for 1 h according to the TRANCE-MRI protocol were excluded. Thirty patients were initially evaluated. One patient was excluded due to possible pregnancy, and another was unable to lie down due to complicated spine disease. Of the 28 patients scheduled for MRI examination, 2 patients were too obese to fit into the scanner, and 1 female patient was unable to complete the scan due to restless legs. There were 16.7% patients (5/30) excluded from this TRANCE-MRI study. All patients received Doppler ultrasonography for evaluating the venous status of their lower extremities before the scheduled TRANCE-MRI. The femoral veins, great saphenous veins, popliteal veins and perforating veins in the calves were checked. Pelvic veins were not included in the Doppler ultrasonography exams. Lymphoscintigraphy with Tc-99 m phytate and CTA were arranged according to the diagnostic indications. The results of DVT in the thigh diagnosed with ultrasonography and TRANCE-MRI were used to calculate the sensitivity, specificity, and accuracy of TRANCE-MRI. Cohen’s kappa coefficient was used to measure the inter-rater agreement between ultrasonography and TRANCE-MRI.

### MRI acquisition

**Fig. 1:**
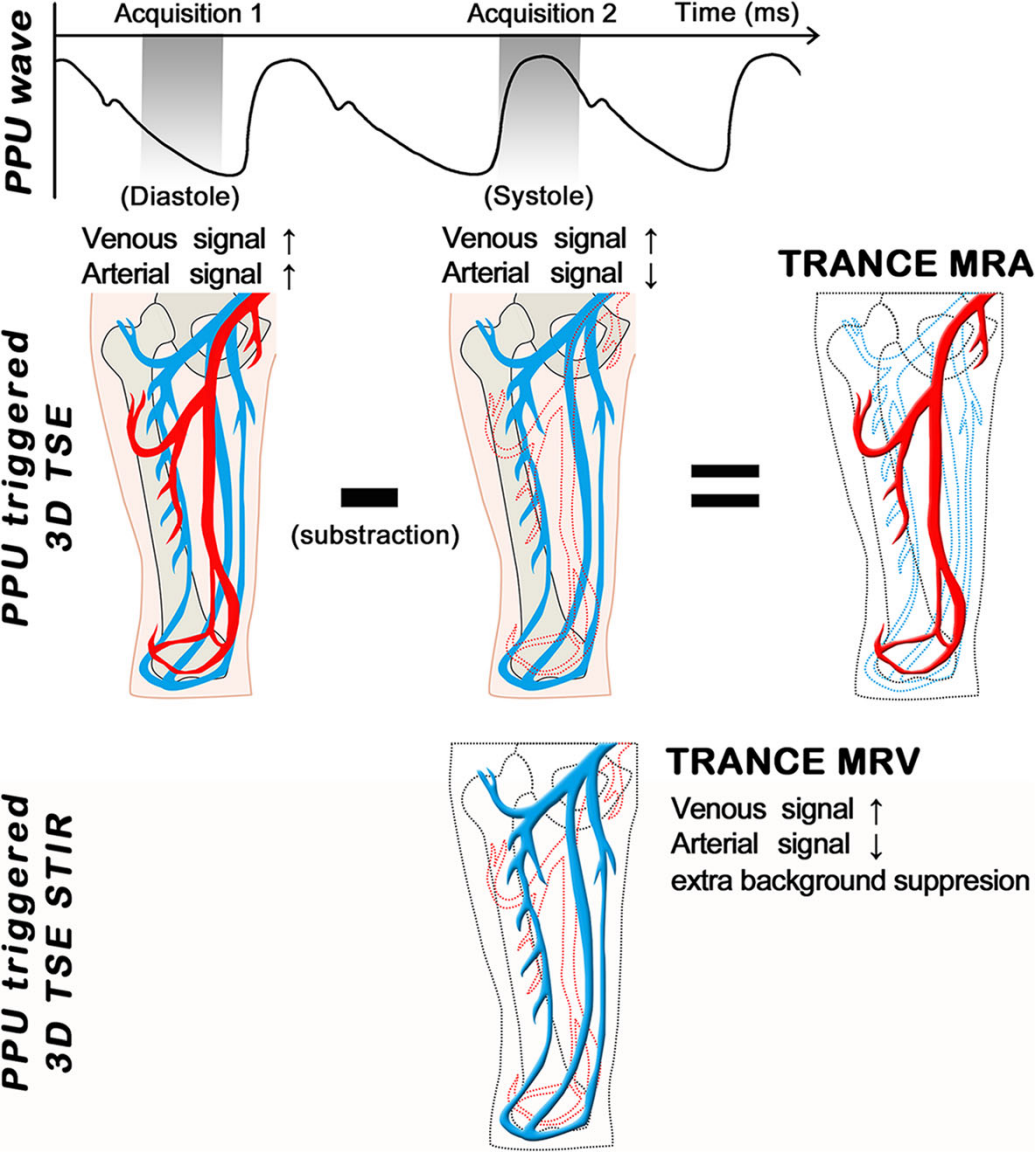
Summarized principle of TRANCE-MRI technique, Patients underwent imaging with a peripheral pulse unit (PPU) trigger. All images of the arterial systems were evaluated by three-dimensional (3D) turbo spin-echo (TSE) at systolic and diastolic periods. During systole, arterial blood flows rapidly and the arteries were black. During diastole, blood flow in the arteries is slow and the arteries were bright. Subtraction of the two phased scans made up a 3D data set with only arteries (MRA). Another image of the venous systems (MRV) was evaluated by 3D TSE Short tau inversion recovery (STIR) during the systolic period. STIR provides additional background suppression because the fat and bones are also suppressed

**Fig. 2:**
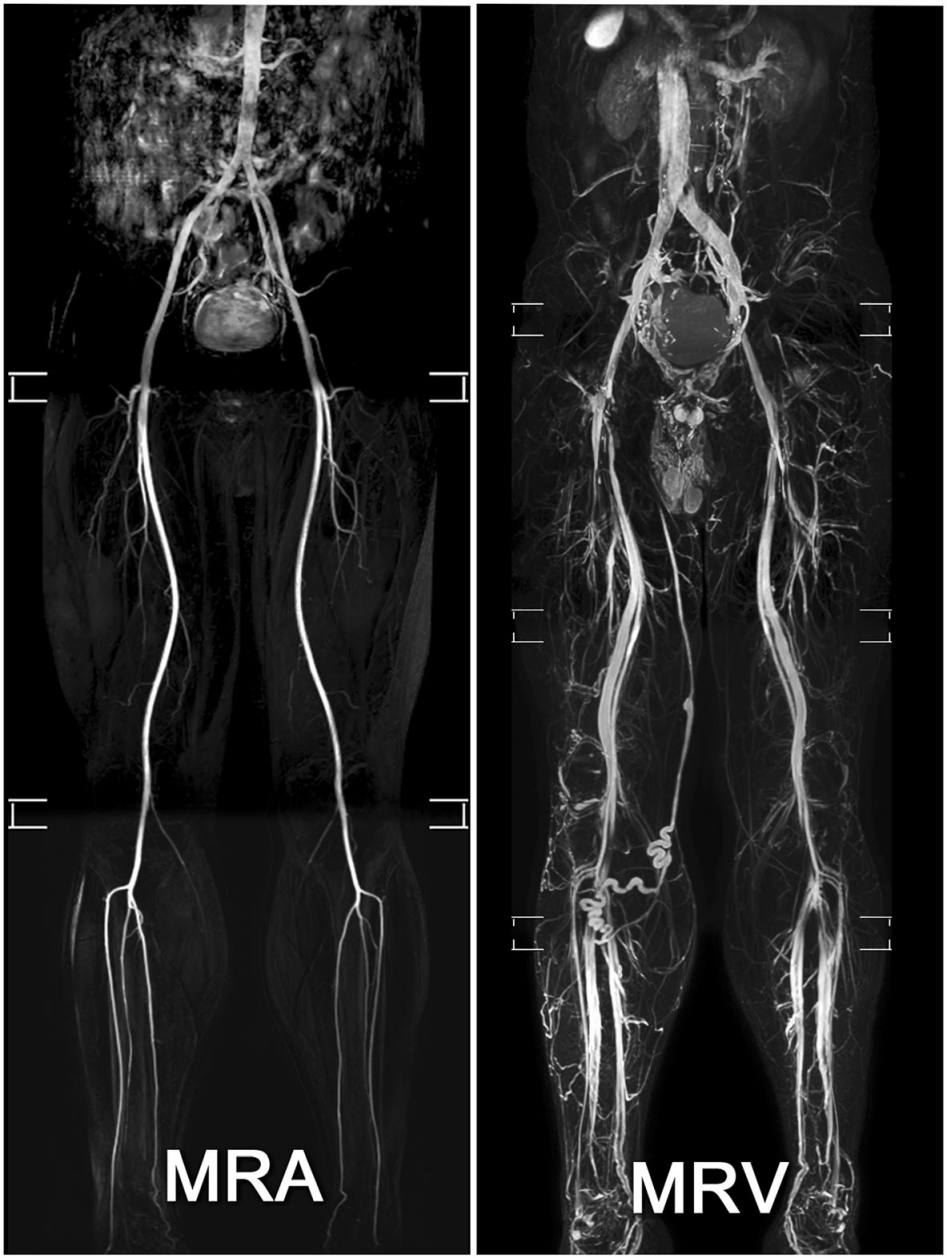
TRANCE MRA and MRV images, TRANCE-MRI was performed on an elder patient. MRA showing arterial patency of bilateral lower extremities. MRV showing a large varicose vein along the right greater saphenous vein

MR imaging was acquired with a 1.5 T MR scanner (Philips Ingenia, Philips Healthcare, Best, the Netherlands). Patients underwent imaging in a supine position and with a peripheral pulse unit trigger. All images of the arterial systems were evaluated by three-dimensional (3D) turbo spin-echo (TSE) at systolic and diastolic periods. In the imaging with the use of TSE TRANCE, the following ranges of parameters were used: repetition time (TR), 1 beat; echo time (TE), shortest; flip angle, 90°; voxel size, 1.7 × 1.7 × 3 mm; and field of view (FOV), 350 × 420. During systole, arterial blood flows rapidly. This causes dephasing of the signal and leads to flow voids; thus, the arteries were black from systolic triggering. During diastole, blood flow in the arteries is slow. The signal does not dephase; thus, the arteries were bright on the diastolic scans. Subtraction of the two phased scans made up a 3D data set with only arteries, as known as magnetic resonance arteriography (MRA). Another image of the venous systems was evaluated by 3D TSE Short tau inversion recovery (STIR) during the systolic period. In the imaging with the TSE STIR TRANCE, the following ranges of parameters were used: TR, 1 beat; TE, 85; inversion recovery delay time, 160; voxel size, 1.7 × 1.7 × 4 mm; and FOV, 360 × 320. STIR provides additional background suppression because the fat and bones are also suppressed. With systolic triggering, the arteries were black. The result was a 3D dataset with only veins, as known as magnetic resonance venography (MRV). A quantitative flow scan was routinely performed to determine the appropriate trigger delay times for systolic and diastolic triggering. All the images were acquired without the use of gadolinium contrast medium. Figure 1 summarizes the principle of the TRANCE technique. The complete TRANCE protocol required 60 min for imaging acquisition, 25 min for MRV, and 35 min for MRA as shown in Fig. 2. The reason for including this time-consuming arterial phase is that the TRANCE technique could evaluate the arteries without contrast-media and probably is less harmful to the patients. Moreover, the leg arterial status in the patients with venous static ulcers is crucial for further compressive therapy to enhance ulcer healing.

## Results

**Fig. 3:**
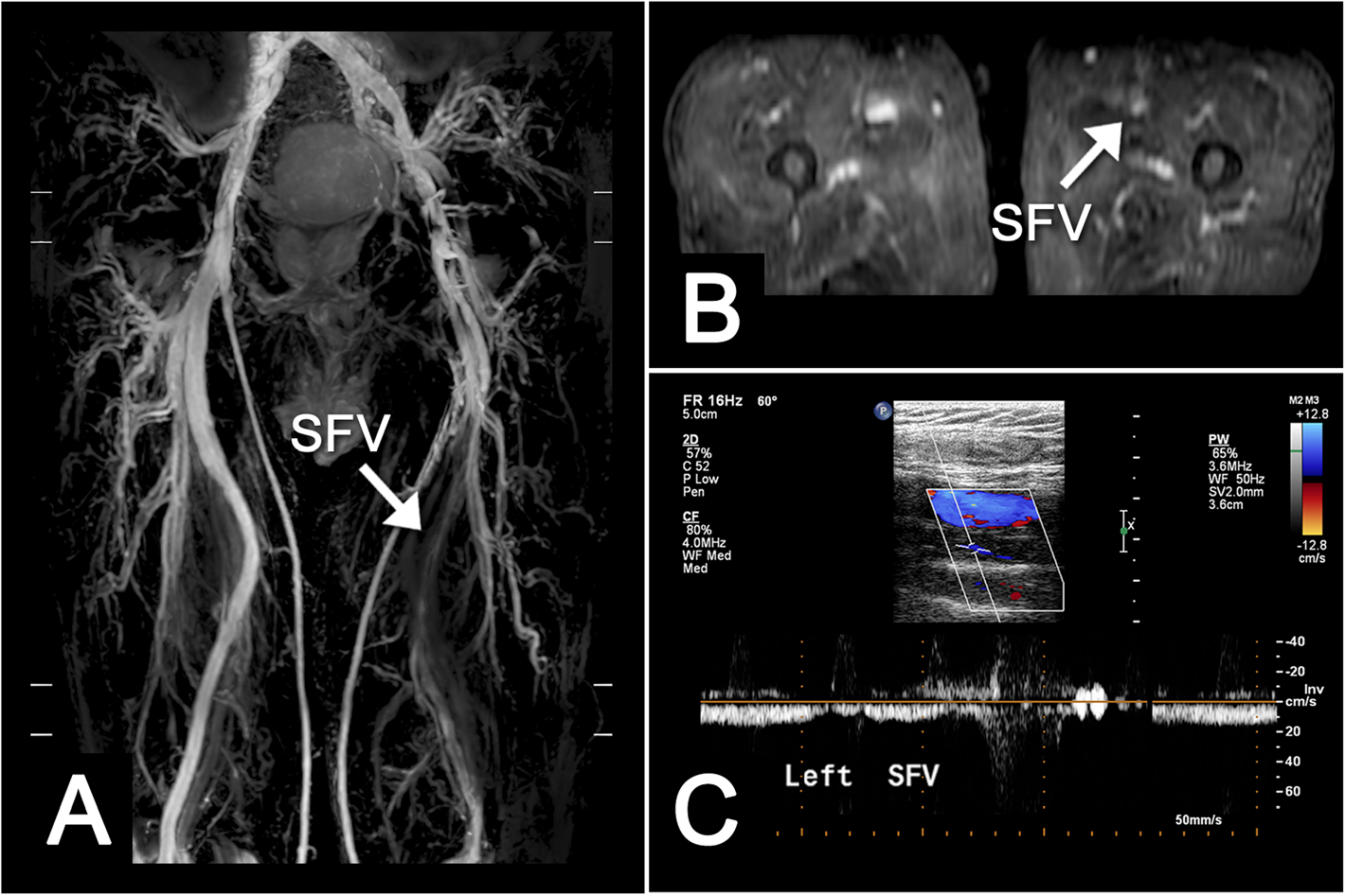
Deep vein thrombosis (DVT), Coronal (**a**) and axial (**b**) images showed incomplete opacification of left superficial femoral vein (SFV), consistent with DVT. Doppler ultrasound also showed the same result (**c**)

A total of 25 patients were enrolled. According to the initial assessment in the vascular wound centre, these patients were classified into five different venous scenarios: deep vein thrombosis (DVT), static ulcer (SU), symptomatic varicose veins (symptomatic VVs), recurrent varicose veins after venous surgery (recurrent VVs) and lymphoedema. The venous scenarios from the vascular wound centre were DVT in 11 patients (44%; Fig. 3), SU in seven patients (28%), symptomatic VVs in three patients (12%), recurrent VVs after surgery in two patients (8%), and possible lymphoedema in two patients (8%). The descriptive characteristics of this population are listed in Table 1. All 25 patients were evaluated by Doppler ultrasonography for venous evaluation and by TRANCE-MRI for both venous and arterial evaluations. Three patients had a CTA scan with contrast media injection. One patient had ultrasonography spected radiation-related lymphoedema and received radionuclide lymphoscintigraphy and a venogram to confirm the diagnosis.

**Table 1:** Descriptive characteristics of 25 participants

Gender-male, No. (%)	19 (76%)
Age, mean ± SD, y	62 ± 13.9
Initial assessment of venous scenarios	
Deep vein thrombosis, No. (%)	11 (44%)
Venous static ulcer, No. (%)	7 (28%)
Symptomatic varicose vein, No. (%)	3 (12%)
Recurrent varicose vein, No. (%)	2 (8%)
Lymphoedema, No. (%)	2 (8%)
History of vascular procedure	
Stripping of GSV, No. (%)	2 (8%)
Truncal ablation of GSV, No. (%)	3 (12%)
Left axillo-femoral arterial bypass graft, No. (%)	1 (4%)
Total hip replacement, No. (%)	1 (4%)
Free flap for left leg crushing injury, No. (%)	1 (4%)
Hysterectomy plus radial therapy, No. (%)	1 (4%)
Imaging evaluation	
TRANCE in venous system, No. (%)	25 (100%)
TRANCE in arterial system, No. (%)	25 (100%)
Doppler ultrasonography in venous system, No. (%)	25 (100%)
Computed tomography angiography, No. (%)	3 (12%)
Radionuclide lymphoscintigraphy, No. (%)	1 (4%)
Conventional venography (for angioplasty), No. (%)	1 (4%)

*GSV* Greater saphenous vein, *TRANCE MR* Triggered angiography non-contrast-enhanced sequence magnetic resonance imaging

The details regarding the age, sex, and TNACE-MRI imaging findings from studies are summarized in Table 2. The sensitivity, specificity and accuracy of TRANCE-MRI were 85.7%, 88/9 and 88%, respectively. The inter-rater agreement for DVT of the thigh between the ultrasonography and TRANCE-MRI results was substantial agreement (Cohen’s kappa κ, 0.72). In the ultrasonography-negative patients, TRANCE-MRI detected four additional cases (16%, 4/25) of DVT; three cases (12%, 3/25) of venous compression caused by pelvic lymphadenopathy, hip prosthesis or knee joint effusion; one case (4%, 1/25) of vena cava anomaly; two cases (8%, 2/25) of occult peripheral artery disease (PAD); and one case (4%, 1/25) of an occluded bypass graft.

**Table 2:** Finds of TRANCE MRI (artery and venous system) and other image studies

No.	Venous Scenario	Age/Sex	TRANCE-MRI	Ultrasound and other image studies
1	Deep vein thrombosis	69/M	DVT in left SFV	US: DVT in left SFV.
2	Deep vein thrombosis	55/M	DVT in left EIV	US & CT: no thrombosis
3	Deep vein thrombosis	59/M	DVT in left PV	US: DVT in left PV
4	Deep vein thrombosis	72/F	DVT in left CFV and SFV	US: DVT in left CFV and SFV.
5	Deep vein thrombosis	57/F	DVT in right EIV	US & CT: no thrombosis
6	Deep vein thrombosis	78/M	DVT in left CFV and SFV	US: DVT in left CFV and SFV.
7	Deep vein thrombosis	74/F	DVT in left CFV, SFV, PV, ATV and PTV; PAOD in left CFA and SFA	US: DVT in left CFV, SFV, PV
8	Deep vein thrombosis	84/M	Both EIVs compression by metastatic lymph nodes; PAD in left ATA	US: no thrombosis
9	Deep vein thrombosis	74/M	Left CFV compression by hip prosthesis with osteomyelitis	US: no thrombosis
10	Deep vein thrombosis	72/F	Left PV compression by knee effusion	US: no thrombosis
11	Deep vein thrombosis	78/M	Double IVC	US: no thrombosis
12	Venous static ulcer	53/M	Varicose veins of SSV territory with large perforators	US: prominent calf perforating varicose veins
13	Venous static ulcer	34/M	Collateral veins from medial calf to medial thigh, with pelvic collateral vein	US: no thrombosis
14	Venous static ulcer	68/M	DVT im left PV and PTV, subcutaneous swelling of left leg	US: venous insufficiency
15	Venous static ulcer	60/M	DVT in right SFV and PV; PAD in right ATA and PTA	US: venous insufficiency
16	Venous static ulcer	78/M	DVT involving from left EIV to left PV	US: DVT in left thigh
17	Venous static ulcer	55/M	no thrombosis; no arterial occlusion	US: no thrombosis
18	Venous static ulcer	65/M	Varicose veins of SSV territory; occluded left axillofemoral bypass graft	US: no thrombosis
19	Symptomatic varicose vein	38/M	Varicose veins of both GSVs territory	US: both GSVs insufficiency.
20	Symptomatic varicose vein	38/M	Varicose veins of right GSV territory	US: right GSV insufficiency.
21	Symptomatic varicose vein	54/F	Varicose vein in left GSV	US: left GSV insufficiency.
22	Recurrent varicose vein	50/F	Varicose veins of both legs	US: DVT in left DFV
23	Recurrent varicose vein	45/M	Varicose veins of right leg	US: no thrombosis
24	Lymphedema	65/M	diffuse subcutaneous swelling; compression of left CIV	US & CT: no thrombosisLymphoscintigraphy: lymphedema
25	Lymphedema	75/M	Subcutaneous swelling of left leg, no thrombosis.	US: no thrombosis.

*ATA* Anterior tibial artery, *ATV* Anterior tibial vein, *CFA* Common femoral artery, *CFV* Common femoral vein, *CIV* Common iliac vein, *CT* Computed tomography, *DFV* Deep femoral vein, *DVT* Deep venous thrombosis, *EIV* External iliac vein, *GSV* Greater saphenous vein, *IVC* Inferior vena cava, *PAD* Peripheral artery disease, *PV* Popliteal vein, *PTV* Posterior tibial vein, *SFA* Superficial femoral artery, *SFV* Superficial femoral vein, *SSV* Small saphenous vein, *US* Ultrasonography

Of the 11 cases of venous DVT, 3 cases (12%, 3/25) were caused by abdominal and pelvic lesions (e.g., pelvic lymphadenopathy, hip prosthesis, and vena cava abnormality), which were difficult to assess with ultrasound alone. Case 8 demonstrated external iliac veins that were compressed by enlarged lymph nodes and were then diagnosed as advanced prostate cancer. In cases 9 and 10, venous congestion was caused by external compression from the hip prosthesis with osteomyelitis and joint effusion. The venous congestion of the lower extremities may have been caused by a double inferior vena cava in case 11 (Additional file 1). Case 18 demonstrated an unhealed wound attributed to graft occlusion of the left axillo-femoral arterial bypass, which could not be detected with ultrasonography alone. Of the 5 cases of symptomatic and recurrent VVs, TRANCE-MRI could yield ultra-clear images, allowing for multiplanar vessel reconstruction that may have been helpful for pre-intervention assessment and planning. Of the 2 cases of lymphoedema, an imaging pattern of diffuse subcutaneous oedema could be detected with TRANCE-MRI. However, if there was no clinical information, it was difficult to distinguish between lymphoedema (case 24) and cellulitis (case 25).

## Discussion

TRANCE is a non-contrast-enhanced MR technique that was first described by Wedeen in 1985 [13]. TRANCE-MRI has been applied in cranial neurologic diseases and arterial diseases; however, few applications of this technique in venous pathology have been found [14–18]. The principle of the TRANCE-MRI technique is that different blood flow velocities will have different signal intensities on the TSE sequence. It can display ultra-clear and background-removed blood vessel images, including arteriography along, venography along, or background-removed arteries and veins. According to our initial experience performing TRANCE-MRI, varicose veins and their territories can be clearly displayed, and venous thrombosis and compression can be found and distinguished, which may be helpful for pre-intervention assessment and planning. Presentation of only the venous structure without the accompanying arterial structure is difficult to achieve on contrast-enhanced MRI or CT because the proper acquisition time is short and variable. This study highlights that TRANCE-MRI can be used as an alternative and objective tool for assessing lower extremity diseases, especially suspected venous pathology.

Doppler ultrasonography is considered the first-line tool for evaluating lower limb swelling. In this present study, inter-rater agreement for DVT of the thigh between the ultrasonography (gold standard) and TRANCE-MRI results was substantial agreement. The sensitivity, specificity and accuracy of TRANCE-MRI were 85.7, 88.9 and 88%, respectively. Therefore, we believe that TRANCE-MRI can be used as an alternative and objective tool for assessing lower extremity diseases, especially suspected venous pathology. Furthermore, in ultrasonography-negative cases, TRANCE-MRI could detect further cases of DVT, venous compression, vena cava anomaly, occult PAD and occluded bypass grafts. Compared with TRANCE-MRI, ultrasonography played a relatively small role in assessing varicose veins of the lower extremities and deep veins of the pelvis and abdomen. We still consider that ultrasound should be used preferentially when assessing venous lesions in the lower extremities because it is non-invasive and cost-effective. If a patient has an abdominal pelvic venous problem or complicated varicose veins, non-contrast-enhanced MRI techniques, such as TRANCE, may be helpful for pre-intervention assessment and planning.

In the present study, we designed a complete MR protocol (total acquisition time, 60 min) for imaging acquisition of all (infra-diaphragmatic) lower extremity arteries (MRA; acquisition time, 35 min) and veins (MRV; acquisition time, 25 min) to fully explore its clinical utility and potential diagnostic value. Thus, this protocol is not suitable for critical and irritable patients and should be modified to reduce the imaging acquisition time in selective patients. In this study, the majority of cases (92%, 23/25) were attributed to venous disease only, and the MRA results were negative. Therefore, we recommend performing a TRANCE-MRV protocol (acquisition time, 25 min) instead of the full protocol (MRV + MRA) in the clinical setting in patients with venous scenarios.

**Fig. 4:**
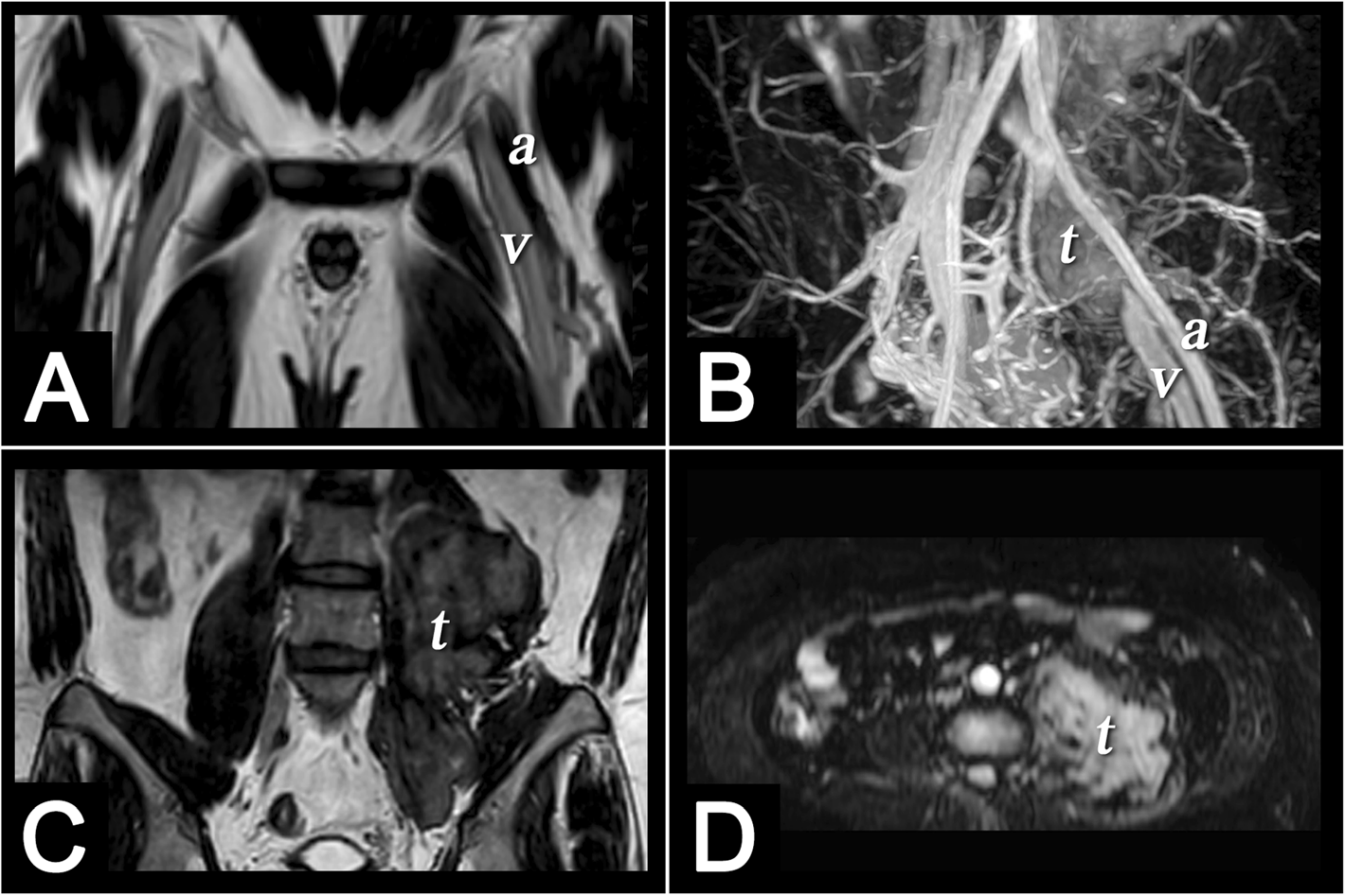
Multiplanar MRI was helpful for comprehensive diagnosis, Our MRI protocol provide coronal and axial images, as well as 3D MRA and MRV images. (**a**) Coronal T2-weighted TSE image of the bilateral inguinal regions and proximal thighs showed high signal intensity of left common femoral vein (v) reflecting slow venous blood flow. In contrast, flow void effect of left common femoral artery (a) reflecting a very high velocity of systolic arterial blood flow. **b** 3D TSE STIR sequence triggered in diastole shows both venous and arterial structures with background subtraction. A retroperitoneal tumor (t) was observed in the left iliopsoas region, causing venous compression but still maintaining arterial patency. Coronal T2-weighted TSE image (**c**) and axial TSE STIR image (**d**) showed comprehensive diagnosis of the retroperitoneal tumor (t)

**Fig. 5:**
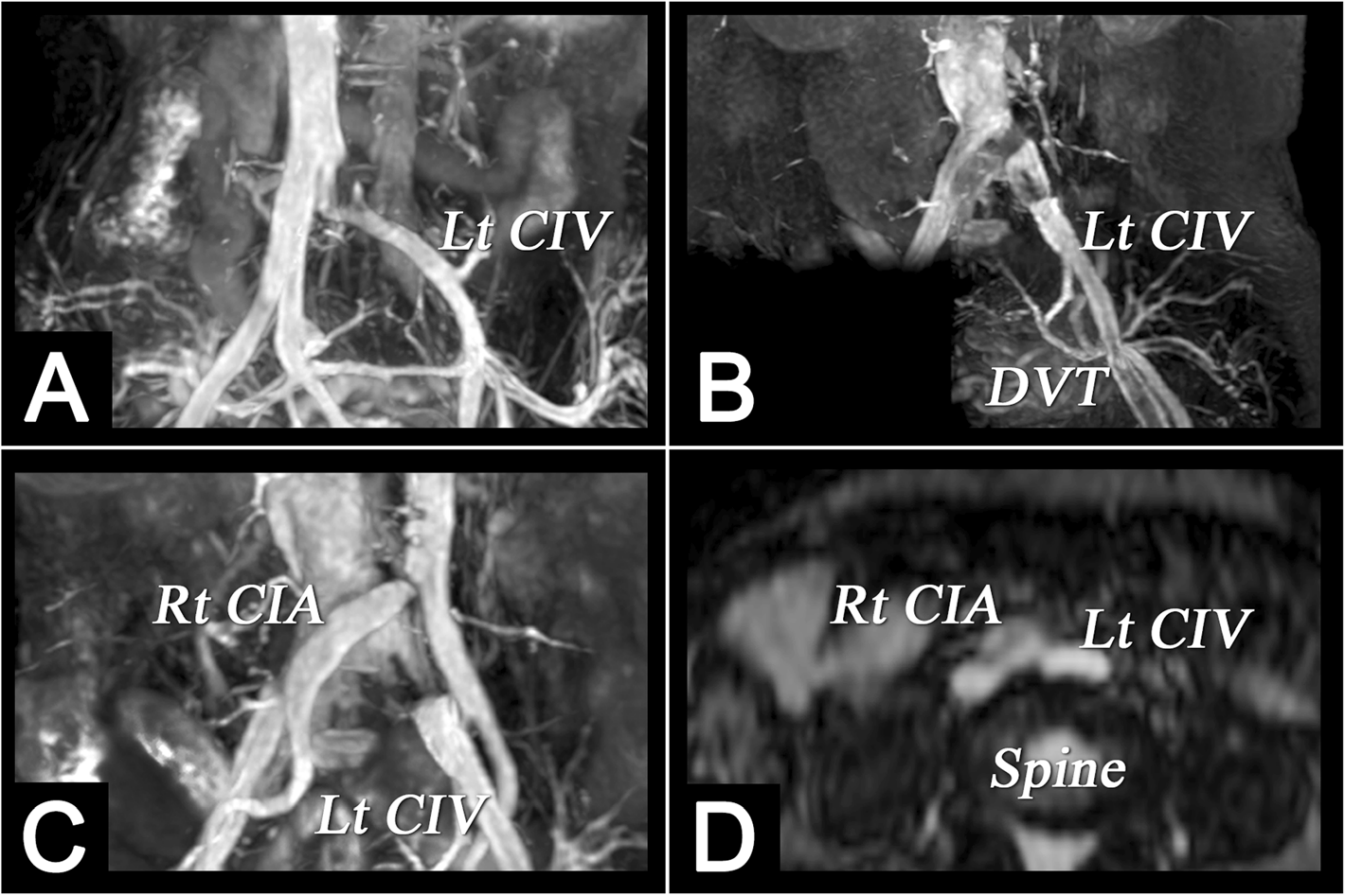
TRANCE-MRV showing interruption of the left common iliac vein and May-Thurner syndrome, (**a**) TRANCE-MRV showed that many subjects had equivocal interruption of the left common iliac vein (CIV), but no venous thrombosis, collateral vessels or related symptoms. This may be because the left CIV is located between the right common iliac artery (CIA) and the spine, which is an anatomically relatively narrow location. **b**-**d** May-Thurner syndrome in a patient with recurrent DVT in the left leg. **b** MRV shows interruption of the left CIV and DVT of left femoral vein. **c**, **d** MRV and MRA show compression of the left CIV against the lumbar vertebrae by the overlying right CIA

In this study, we did not specifically describe how to distinguish between acute and chronic thromboses. Distinguishing acute from chronic DVT is a potential advantage of MRI, with irregular wall thickening in the presence of collaterals and a diminutive lumen suggestive of chronic DVT. Our MRI protocol provides coronal and axial images, as well as 3D MRA and MRV images. We used the original unremoved background image to examine possible tumours or other causes of compression for all vascular lesions (Fig. 4). TRANCE-MRV showed that many subjects had equivocal interruption of the left common iliac vein but no venous thrombosis, collateral vessels or related symptoms. This may be because the left common iliac vein is located between the right common iliac artery and the spine, which is an anatomically and relatively narrow location (Fig. 5).

Several advantages of TRANCE-MRI application in venous pathology in the lower extremities exist. First, TRANCE-MRI provides not only images of the arteries and veins in the lower extremities but also information on the pelvis and abdomen, which is valuable in patients with a venous scenario of DVT. DVT may be mistaken as external compression of the pelvic vessels. Moreover, it is notorious as a sign of occult malignancies. Among the 11 patients with a venous scenario of DVT, four of them (36.4%) had no DVT and the symptoms were attributed to malignancy, external compression by degenerated hip prosthesis, external compression by knee effusion, and congenital anomaly. Second, the thrombi and collateral veins can be clearly outlined, including deep femoral vein that might be difficult to detect by ultrasonography. This may be helpful in catheter-based thrombolytic therapy and rescue therapy in recurrent VV after truncal ablations of GSV. Finally, because TRANCE-MRI has no radiation and does not use contrast media, it is safe for patients with impaired renal function.

We did learn of some drawbacks to TRANCE-MRI according to this study. First, TRANCE-MRI of the venous system may cause false-positive results in the left iliac vessels, which could be attributed to the complex anatomy and overlapping of the vessels with different directions of blood flow. Other observations, such as increasing the diameter and number of collateral veins, constant filling defects, and the application of intravascular ultrasound, may decrease the risk of incorrect diagnosis. Second, this TRANCE-MRI protocol requires 60 min for imaging acquisition, 25 min for MRV, and 35 min for MRA. Thus, it is not suitable for critical and irritable patients. We suggest that the MRI protocol should be determined according to the patient’s condition, and it is not necessary to perform the whole TRANCE-MRI protocol. Finally, TRANCE-MRI is expensive and not widely used at our institution.

The major limitation of this investigation was that it was a non-randomized study with few patients. This study was also limited by a lack of comparison of inter-observer variability and adequate validation with other imaging studies. However, we attempted to identify the values and pitfalls of TRANCE-MRI in venous pathology. This was the first prospective study to apply TRANCE-MRI for assessing venous pathology in the lower extremities. Further evaluation of the pelvic/abdominal assessment and accuracy of TRANCE MRI is needed before implementing versatile clinical applications. TRANCE-MRI may provide more useful information regarding optimal therapeutic protocols for the treatment of complicated vascular diseases.

## Conclusion

TRANCE-MRI can be used as an alternative and objective tool for assessing lower extremity diseases, especially suspected venous pathology. Compared with ultrasonography, TRANCE-MRI plays a better role in assessing varicose veins of the lower extremities and deep veins of the pelvis and abdomen. However, false-positive results may occur in the left common iliac vein of elderly patients. Finally, occult arterial occlusion rarely occurs in patients with suspected lower extremity venous disease. Therefore, we recommend performing the TRANCE-MRV protocol (acquisition time, 25 min) instead of the full protocol (MRV + MRA) in the clinical setting in patients with venous scenarios.

## Supplementary information

**Additional file 1.** TRANCE-MRV showing double inferior vena, The scan field of this MRI protocol was up to abdomen. Venous congestion due to abdominal lesions such as malignancy, external compression, and congenital anomaly were able to be evaluated.

## Data Availability

The dataset(s) supporting the conclusions of this article is(are) included within the article (and its additional file(s)).

## Abbreviations

3D: Three-dimensional
CT: Computed tomography
CTA: Computed tomography angiography
DVT: Deep venous thrombosis
FOV: Field of view
IRB: Institutional review board
MRA: Magnetic resonance arteriography
MRI: Magnetic resonance imaging
MRV: Magnetic resonance venography
NSF: Nephrogenic systemic fibrosis
PAD: Peripheral artery disease
STIR: Short tau inversion recovery
SU: Static ulcer
TE: Echo time
TOF: Time-of-flight
TR: Repetition time
TRANCE: Triggered angiography non-contrast enhanced
TSE: Turbo spin-echo
US: Ultrasonography
VV: Varicose vein
